# Altered trends in carbon uptake in China's terrestrial ecosystems under the enhanced summer monsoon and warming hiatus

**DOI:** 10.1093/nsr/nwz021

**Published:** 2019-02-17

**Authors:** Honglin He, Shaoqiang Wang, Li Zhang, Junbang Wang, Xiaoli Ren, Lei Zhou, Shilong Piao, Hao Yan, Weimin Ju, Fengxue Gu, Shiyong Yu, Yuanhe Yang, Miaomiao Wang, Zhongen Niu, Rong Ge, Huimin Yan, Mei Huang, Guoyi Zhou, Yongfei Bai, Zongqiang Xie, Zhiyao Tang, Bingfang Wu, Leiming Zhang, Nianpeng He, Qiufeng Wang, Guirui Yu

**Affiliations:** 1 Synthesis Research Center of China's Ecosystem Research Network, Key Laboratory of Ecosystem Network Observation and Modeling, Institute of Geographic Sciences and Natural Resources Research, Chinese Academy of Sciences, Beijing 100101, China; 2 College of Resources and Environment, University of Chinese Academy of Sciences, Beijing 100190, China; 3 Sino-French Institute for Earth System Science, College of Urban and Environment Sciences, Peking University, Beijing 100871, China; 4 Key Laboratory of Alpine Ecology and Biodiversity, Institute of Tibetan Plateau Research, CAS Center for Excellence in Tibetan Plateau Earth Science, Chinese Academy of Sciences, Beijing 100085, China; 5 National Meteorological Center, China Meteorological Administration, Beijing 100081, China; 6 International Institute for Earth System Science and Jiangsu Provincial Key Laboratory of Geographic Information Science and Technology, Nanjing University, Nanjing 210023, China; 7 Key Laboratory of Dryland Agriculture, MOA, Institute of Environment and Sustainable Development in Agriculture, Chinese Academy of Agricultural Sciences, Beijing 100081, China; 8 School of Geography, Geomatics, and Planning, Jiangsu Normal University, Xuzhou 221116, China; 9 State Key Laboratory of Vegetation and Environmental Change, Institute of Botany, Chinese Academy of Sciences, Beijing 100093, China; 10 University of Chinese Academy of Sciences, Beijing 100049, China; 11 Key Laboratory of Vegetation Restoration and Management of Degraded Ecosystems, South China Botanical Garden, Chinese Academy of Sciences, Guangzhou 510650, China; 12 Department of Ecology, College of Urban and Environmental Science, Key Laboratory for Earth Surface Processes of the Ministry of Education, Peking University, Beijing 100871, China; 13 Institute of Remote Sensing and Digital Earth, Chinese Academy of Sciences, Beijing 100094, China

**Keywords:** climate change, Asian summer monsoon, warming hiatus, biogeochemical modeling, ecosystem carbon dynamics

## Abstract

The carbon budgets in terrestrial ecosystems in China are strongly coupled with climate changes. Over the past decade, China has experienced dramatic climate changes characterized by enhanced summer monsoon and decelerated warming. However, the changes in the trends of terrestrial net ecosystem production (NEP) in China under climate changes are not well documented. Here, we used three ecosystem models to simulate the spatiotemporal variations in China's NEP during 1982–2010 and quantify the contribution of the strengthened summer monsoon and warming hiatus to the NEP variations in four distinct climatic regions of the country. Our results revealed a decadal-scale shift in NEP from a downtrend of –5.95 Tg C/yr^2^ (reduced sink) during 1982–2000 to an uptrend of 14.22 Tg C/yr^2^ (enhanced sink) during 2000–10. This shift was essentially induced by the strengthened summer monsoon, which stimulated carbon uptake, and the warming hiatus, which lessened the decrease in the NEP trend. Compared to the contribution of 56.3% by the climate effect, atmospheric CO_2_ concentration and nitrogen deposition had relatively small contributions (8.6 and 11.3%, respectively) to the shift. In conclusion, within the context of the global-warming hiatus, the strengthening of the summer monsoon is a critical climate factor that enhances carbon uptake in China due to the asymmetric response of photosynthesis and respiration. Our study not only revealed the shift in ecosystem carbon sequestration in China in recent decades, but also provides some insight for understanding ecosystem carbon dynamics in other monsoonal areas.

## INTRODUCTION

In recent years, China has become the largest carbon emitter in the world [1]. Meanwhile, the terrestrial ecosystems of China are functioning as strong carbon sinks and absorbing a significant amount of carbon dioxide (CO_2_) from the atmosphere, which can partly offset the industrial carbon emissions [2,3]. Therefore, the carbon cycle of China's terrestrial ecosystems is an important component of the global carbon budget. Modulated by the East Asian monsoon, the carbon sink of China's terrestrial ecosystems is particularly sensitive to climate changes [4] and exhibits remarkable spatial and temporal variability [5]. Over the past decade, China has experienced a marked shift in climate-change trends. The strengthening of the East Asian summer monsoon has resulted in a dramatic change in the spatial pattern of precipitation, with increased rainfall in North China and reduced rainfall in South China [6]. Moreover, the warming trend has substantially slowed since 1998 [7], which is at pace with the worldwide deceleration in warming known as the global-warming hiatus [8,9].

These changes in the precipitation and temperature regimes could have profound implications for carbon cycles. Studies have suggested that a shift from an increasing trend to a decreasing trend in terrestrial net ecosystem production (NEP) in China may occur in the wake of accelerated climate warming in the twentieth century [4,10]. However, little is known about the responses of terrestrial NEP in China to the dramatic changes in the climate regime that occurred at the beginning of the twenty-first century. Although recent global-scale analyses have shown enhanced terrestrial carbon uptake during the last decade with rising atmospheric CO_2_ concentration and climate changes [11–13], the underlying mechanism is still a matter of debate. The change of terrestrial NEP in China in the latter half of the twentieth century has been well documented in previous studies [2,3,10,14,15]. However, several key questions remain unanswered under the ‘new normal’ of climate changes in the first decade of the twenty-first century. For example, will the terrestrial ecosystems of China continue to serve as a carbon sink? To what extent will the terrestrial NEP respond to current climate changes? How will the changes in NEP vary among regions? Addressing these questions is of great importance not only for improving the predictions of terrestrial carbon balance in China under the threat of future climate changes, but also for providing more accurate information to policymakers.

**Figure 1.**
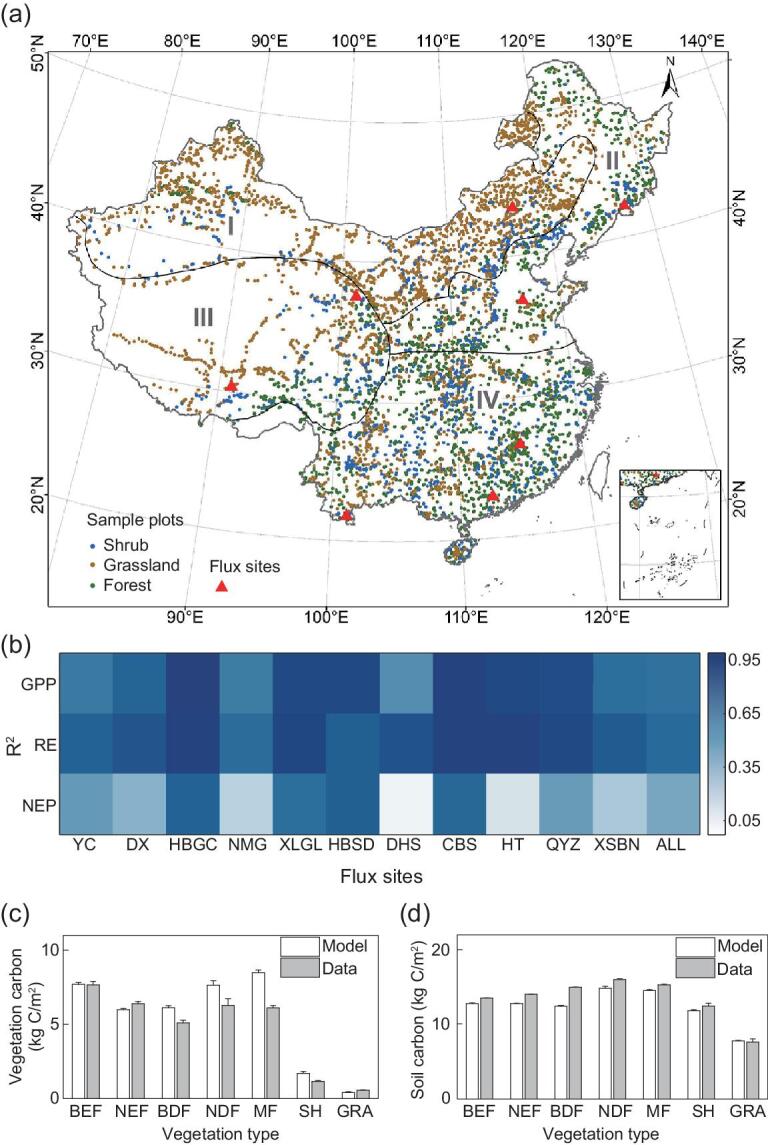
Validation of the ensemble mean of modeled carbon fluxes and storages. (a) Spatial distribution of 11 984 field plots and 11 eddy-covariance flux towers across China. The observational data from these locations were used to validate the models. The four climatic regions are temperate continental (I), temperate monsoonal (II), high-cold Tibetan Plateau (III) and subtropical–tropical monsoonal (IV). (b) Validation of modeled gross primary production (GPP), ecosystem respiration (RE) and net ecosystem production (NEP) against the observational data at 11 flux tower sites. These 11 sites are Yucheng cropland (YC), Dangxiong alpine steppe-meadow (DX), Haibei alpine shrub-meadow (HBGC), Inner Mongolia temperate steppe (NMG), Xilinguole grassland (XLGL), Haibei alpine swamp (HBSD), Dinghushan evergreen mixed forest (DHS), Changbaishan temperate mixed forest (CBS), Huitong evergreen needleleaf forest (HT), Qianyanzhou evergreen needleleaf forest (QYZ), Xishuangbanna evergreen broadleaf forest (XSBN) and all sites together (ALL). The goodness of fit was determined using the coefficient of determination (*R*^2^). (c) Comparison of the modeled and observed vegetation carbon storages among the different vegetation types. (d) Comparison of the modeled and observed soil carbon storages among the various vegetation types. The vegetation types are broadleaf evergreen forest (BEF), needleleaf evergreen forest (NEF), broadleaf deciduous forest (BDF), needleleaf deciduous forest (NDF), mixed forest (MF), shrubland (SH) and grassland (GRA). The error bars in panels (c) and (d) represent the standard error.

Under the Carbon Budget Special Project [16], we obtained *in situ* volumetric observational data, including 74 site years of eddy-covariance data from 11 flux towers of the Chinese Terrestrial Ecosystem Flux Observation and Research Network (ChinaFLUX) [17] and carbon stock data of 11 984 plots with geographic coordinates (Fig. 1a) from the field survey data across China [18]. These data provide a unique opportunity to validate and parameterize carbon-cycle models. In this study, three ecosystem models (CEVSA2, BEPS and TEC; see Supplementary Text 1 and Supplementary Table 1, available as Supplementary Data at *NSR* online) that have been extensively evaluated (see Supplementary Table 2, available as Supplementary Data at *NSR* online) were validated and parameterized using these newly obtained data (Fig. 1b–d). These models were then used to simulate the spatial and temporal variations in terrestrial NEP in China from 1982 to 2010. Furthermore, using a map of climatic regions in China [19], we classified the country into four climatic regions (i.e. temperate continental, temperate monsoonal, high-cold Tibetan Plateau and subtropical–tropical monsoonal) to investigate responses of the terrestrial NEP to climate changes in different climatic regions. Our results revealed a climate-dominated shift in the terrestrial NEP trend in China in recent decades and highlighted the importance of monsoonal precipitation to this shift in the context of warming hiatus.

**Table 1.** Trends of net ecosystem production (NEP) in four climatic regions and throughout China. A positive trend in NEP indicates an increasing sink and a negative trend indicates a decreasing sink.

	1982–2000			2000–10		
Climatic region	Trend (Tg C/yr^2^)	*R* ^2^	*P-*value	Trend (Tg C/yr^2^)	*R* ^2^	*P*-value
Temperate continental (I)	–1.03	0.06	0.293	3.08	0.24	0.128
Temperate monsoonal (II)	–3.40	0.18	0.072	8.77	0.45	0.023
High-cold Tibetan Plateau (III)	0.22	0.02	0.579	–0.38	0.02	0.675
Subtropical–tropical monsoonal (IV)	–1.74	0.08	0.231	2.75	0.03	0.616
China	–5.95	0.17	0.080	14.22	0.32	0.069

**Table 2.** Sensitivity of gross primary production (GPP), ecosystem respiration (RE) and net ecosystem production (NEP) to temperature and precipitation during 1982–2010, as calculated by Equation 1 in the ‘Materials and methods’ section.

	Temperature (Tg C/°C)			Precipitation (Tg C/100 mm)		
Climatic region	GPP	RE	NEP	GPP	RE	NEP
Temperate continental (I)	2.9	11.0	–8.0	119.3	56.5	62.6
Temperate monsoonal (II)	17.7	27.8	–10.1	116.2	58.8	57.4
High-cold Tibetan Plateau (III)	16.2	21.1	–4.9	14.5	13.4	1.1
Subtropical–tropical monsoonal (IV)	78.1	119.8	–41.8	10.2	–6.8	17.0
China	70.5	132.1	–61.6	252.4	135.5	116.8

## RESULTS

### The magnitude and trends of national NEP

**Figure 2.**
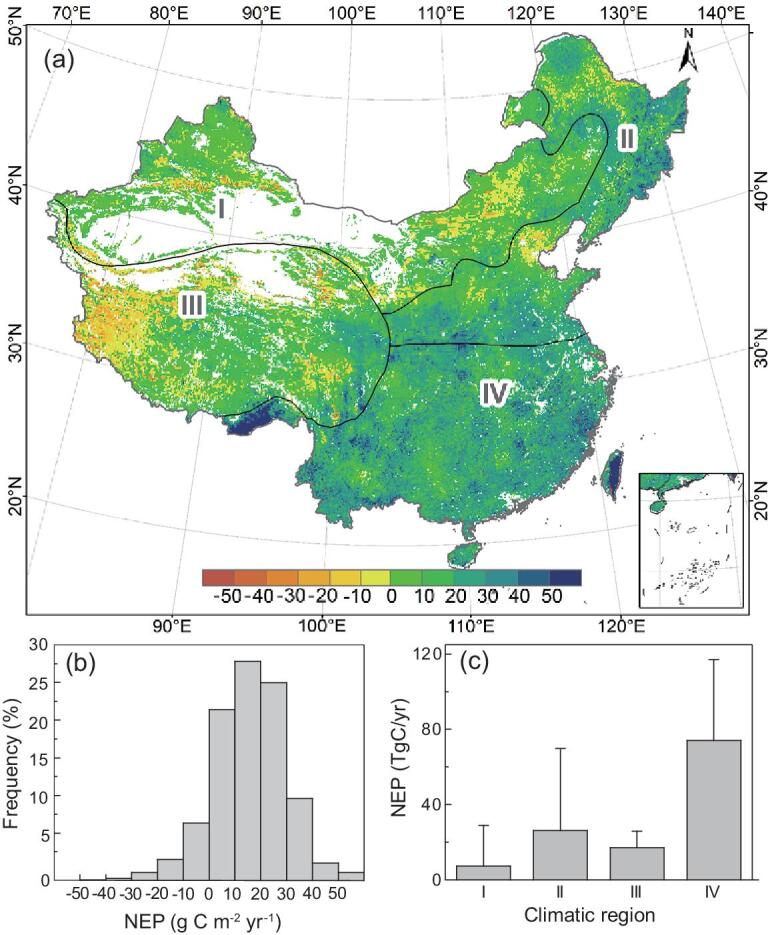
Ensemble mean of modeled net ecosystem production (NEP) in China averaged during 1982–2010. (a) Spatial pattern of the NEP, (b) frequency distribution of the NEP and (c) average NEP in the four climatic regions. The four climatic regions are temperate continental (I), temperate monsoonal (II), high-cold Tibetan Plateau (III) and subtropical–tropical monsoonal (IV). A positive NEP value indicates a carbon sink and a negative value indicates a carbon source.

The ensemble mean of the modeled NEP in China's terrestrial ecosystems showed a large spatial heterogeneity over 1982–2010, with positive values (net carbon sinks) in most areas in the east half of the country and negative values (net carbon sources) in the west parts of the country, especially in the Tibetan Plateau and Inner Mongolia (Fig. 2a). The positive values of NEP covered about 71.8% of the land area (Fig. 2b). Overall, terrestrial ecosystems in China sequestrated 0.118 ± 0.079 Pg C/yr during 1982–2010, of which 0.069 Pg C/yr (58.7%) were from the subtropical–tropical monsoonal region, 0.025 Pg C/yr (21.0%) from the temperate monsoonal region and only small amount from Tibetan Plateau (0.017 Pg C/yr, 14.3%) and temperate continental region (0.007 Pg C/yr, 6.0%) (Fig. 2c).

**Figure 3.**
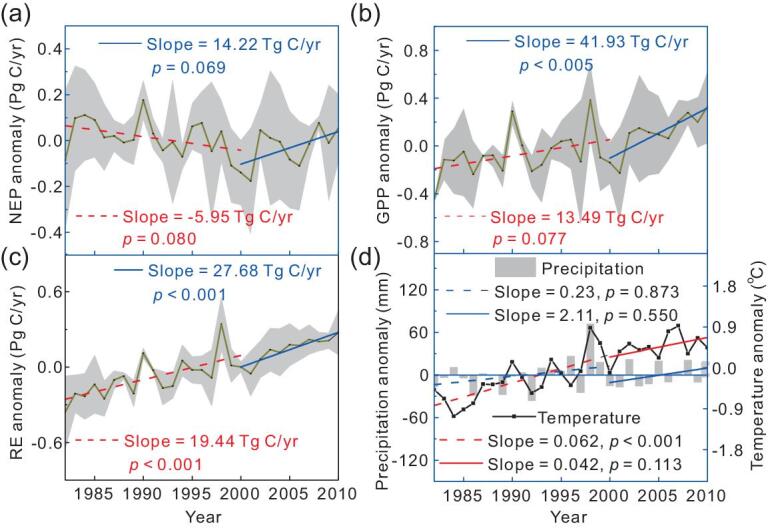
Anomalies of carbon budgets in China's terrestrial ecosystems during 1982–2000 and 2000–10, along with anomalies of climate data. (a) Net ecosystem production (NEP), (b) gross primary production (GPP), (c) ecosystem respiration (RE) and (d) summer precipitation and mean annual temperature. The brown lines and shaded areas in panels (a), (b) and (c) indicate the ensemble mean and 95% confidence interval of the data. The solid and dashed lines delineate the trend determined by linear regression. A positive NEP value indicates a carbon sink.

Interestingly, terrestrial NEP of China showed a decadal-scale shift from a downward trend of −5.95 Tg C/yr^2^ (reduced sink) during 1982–2000 to an upward trend of 14.22 Tg C/yr^2^ (enhanced sink) during 2000–10 (Fig. 3a). The growth rate of gross primary production (GPP) in 2000–10 was 2.1 times higher than that in 1982–2000 (41.93 vs 13.49 Tg C/yr^2^; Fig. 3b), whereas the rate of ecosystem respiration (RE) increased by only 42.4% compared with that of 1982–2000 (27.68 vs 19.44 Tg C/yr^2^; Fig. 3c). This asymmetric increase in GPP and RE led to an overall increase in NEP in the recent decade (2000–10), during which the precipitation trend increased and the warming trend decreased, compared to the first two decades (Fig. 3d).

### NEP trends and the climatic attribution in different regions

**Figure 4.**
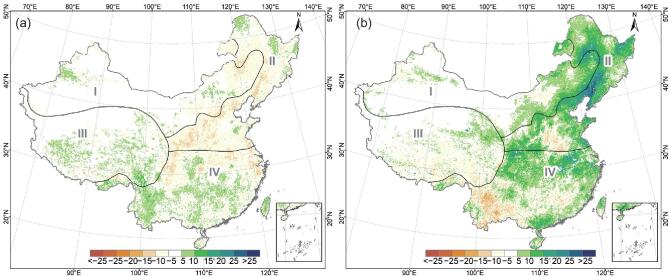
Spatial distribution of the trends in net ecosystem production (NEP) in China during (a) 1982–2000 and (b) 2000–10. Four climatic regions are illustrated to compare the differences in the NEP trends in different regions in the two periods. For details on climatic regions I, II, III and IV, see Fig. 1.

The trend of the modeled NEP varied across the climatic regions during the two periods (Fig. 4 and Table 1). It is noteworthy that the monsoonal areas dominated the NEP trend (Table 1). Specifically, in the first two decades (1982–2000), the modeled NEP trend in the temperate monsoonal region (−3.40 Tg C/yr^2^) accounted for 57.1% of the downward trend in China's NEP, whereas that in the subtropical–tropical monsoonal region accounted for 29.3% (−1.74 Tg C/yr^2^) of this downward trend. On the other hand, in the last decade (2000–10), the temperate monsoonal region contributed 61.7% (8.77 Tg C/yr^2^) to the total country's NEP trend and the subtropical–tropical monsoonal region contributed 19.3% (2.75 Tg C/yr^2^) to this upward trend. Compared to the monsoonal areas, the other two climatic regions played minor roles in the contribution to the national NEP trend.

The multiple regression analysis of the modeled NEP versus climate variables (i.e. temperature and precipitation) showed that the country's NEP decreased with increasing temperature because of the larger effect of warming on RE than on GPP (132.1 vs 70.5 Tg C/°C) and increases in precipitation enhanced NEP because of the greater stimulatory effect of water availability on GPP than on RE (252.4 vs 135.5 Tg C/100 mm) (Table 2). The sensitivity of NEP to temperature and precipitation also varied across the climatic regions. The NEP was more sensitive to precipitation in the temperate monsoonal region (57.4 Tg C/100 mm) and the continental region (62.6 Tg C/100 mm) than the other two climatic regions, due likely to the water limitation during the growing season of ecosystems in the temperate areas. At the same time, the NEP was most sensitive to temperature in the subtropical–tropical monsoonal region (−41.8 Tg C/°C).

**Figure 5.**
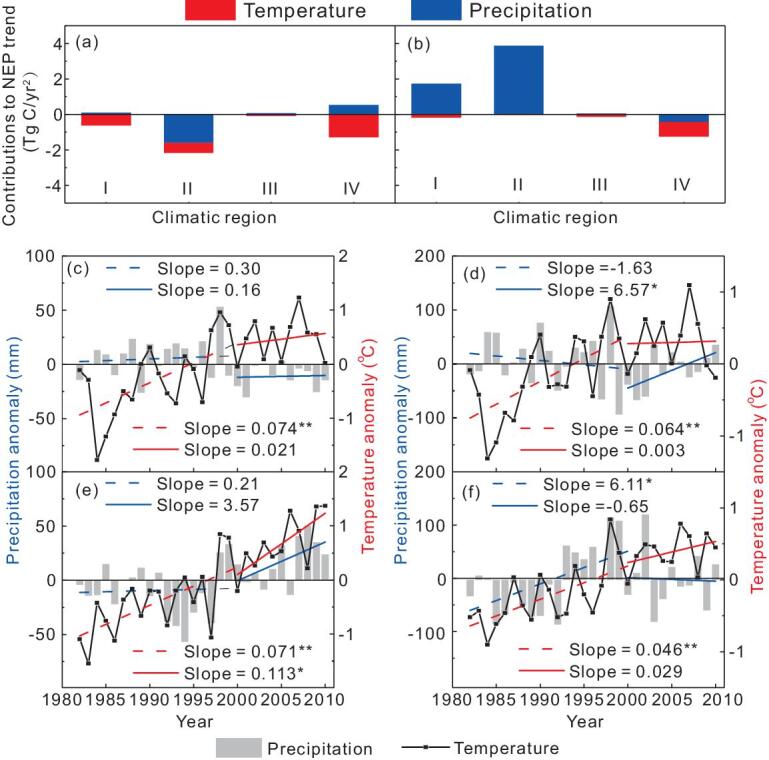
Contribution of precipitation and temperature to the trend in net ecosystem production (NEP) during (a) 1982–2000 and (b) 2000–10, and changes in anomalies of annual temperature and summer precipitation in four climatic regions during 1982–2010. The contribution of precipitation and temperature to NEP trends is calculated by Equation 3 in the ‘Materials and methods’ section; (c) temperate continental, (d) temperate monsoonal, (e) high-cold Tibetan Plateau and (f) subtropical–tropical monsoonal climatic regions.

The trends of the modeled NEP induced by climate changes in different regions, as shown in Fig. 5a and b, were determined by the sensitivity of NEP to temperature and precipitation (Table 2) as well as their variations (Fig. 5c–f). In the temperate continental climatic region, the NEP decreased at a rate of −1.03 Tg C/yr^2^ during 1982–2000 (Table 1). The NEP trend attributed to progressive warming (−0.62 Tg C/yr^2^) contributed 60.5% to the change in this region (Fig. 5c), although the sensitivity of NEP to temperature was relatively low (−8.0 Tg C/°C). The NEP increased at a rate of 3.08 Tg C/yr^2^ during 2000–10 (Table 1) and around 56.0% (1.72 Tg C/yr^2^) of this change was attributed to precipitation due to the high sensitivity of NEP to precipitation (Table 2) and the concurrent wetting trend (Fig. 5c).

In the temperate monsoonal region, the NEP was positively correlated with precipitation because photosynthesis showed a greater response to precipitation than RE (116.2 vs 58.8 Tg C/100 mm; see Table 2). In 1982–2000, precipitation declined progressively (Fig. 5d), which contributed 47.3% (−1.61 Tg C/yr^2^; Fig. 5a) to the decreasing trend of NEP in this region. However, during 2000–10, summer precipitation increased significantly (6.57 mm/yr, *p* < 0.05) (Fig. 5d), which caused a rise in NEP of 3.87 Tg C/yr^2^ (Fig. 5b) that contributed 44.2% to the NEP increase in this region. In the high-cold Tibetan Plateau region, NEP decreased slightly during the year of 2000–10, which was caused by a trade-off between an increase in NEP (0.05 Tg C/yr^2^) due to increasing precipitation and a decrease in NEP (−0.13 Tg C/yr^2^) due to warming (Fig. 5e).

In the subtropical–tropical monsoonal region, NEP was negatively correlated with temperature, because temperature variability had a greater effect on RE than on photosynthesis (119.8 vs 78.1 Tg C/°C; Table 2). During 1982–2000, the decreasing NEP attributed to the progressive warming (−1.29 Tg C/yr^2^; Fig. 5a) accounted for 73.9% of the negative NEP trend in this region. The warming rate declined from 0.046°C/yr (*p* = 0.003) in the period of 1982–2000 to 0.029°C/yr (*p* = 0.247) in the period of 2000–10 (Fig. 5f). Because of the high sensitivity of NEP to temperature in this region, the warming hiatus reduced the negative NEP trend to −0.82 Tg C/yr^2^ in the period of 2000−10 (Fig. 5b), suggesting that the warming hiatus has mitigated the NEP downward trend. Despite the decrease in precipitation, the NEP in this region increased at a rate of 2.75 Tg C/yr^2^ during 2000−10 (Table 1), which was likely caused by the positive effects of nitrogen deposition [20] and afforestation [21,22] (see next section).

### Contributions of different environmental factors to NEP trends

**Figure 6.**
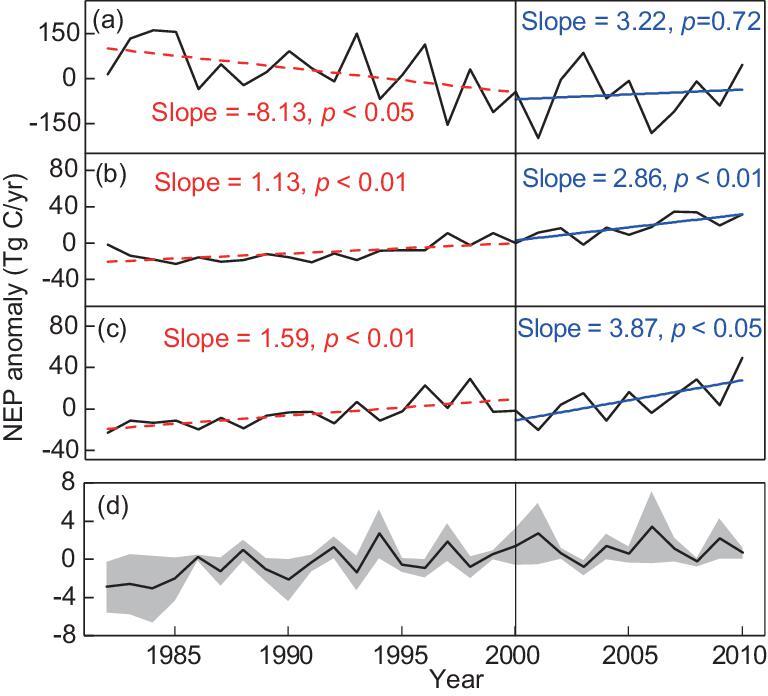
Effects of multiple environmental factors on the modeled net ecosystem production (NEP) in China. (a) Climate, (b) atmospheric CO_2_ concentration and (c) nitrogen deposition. (d) Sensitivity of NEP to changes in land-cover data (1990, 2000 and 2010). The dashed and solid lines in (a), (b) and (c) represent the trends determined by linear regression during 1982–2000 and 2000–10, respectively. The solid line and shaded area in (d) represents the mean and 95% confidence interval, respectively, of the NEP variations using land-cover data in 1990, 2000 and 2010.

The simulation experiments (see ‘Materials and methods’ section) revealed that climate changes were the primary factor that led to an altered NEP trend in the terrestrial ecosystems of China during 1982−2010 (Fig. 6). The trend of modeled NEP induced by climate changes shifted from negative (–8.13 Tg C/yr^2^) during 1982−2000 to positive (3.22 Tg C/yr^2^) during 2000−10 (Fig. 6a). This change accounted for 56.3% of the shift in the NEP trends of China between the two periods. Increasing atmospheric CO_2_ concentration and nitrogen deposition consistently enhanced the NEP during 1982–2010, with a contribution of 1.13 and 1.59 Tg C/yr^2^ during 1982−2000 and 2.86 and 3.87 Tg C/yr^2^ during 2000−10 (Fig. 6b and 5c), respectively. The increase in NEP trends attributed to these two factors contributed 8.6 and 11.3% to the shift in the trend of carbon uptake, respectively. In contrast, the NEP varied by 5.09% when land-cover data in 1990, 2000 and 2010 (Fig. 6d) were used, indicating the minor impact of land-cover data on the national terrestrial carbon sink.

## DISCUSSION

Carbon uptake in China's terrestrial ecosystems accounted for 5.2% of the global carbon sink (2.29 Pg C/yr during 1982–2010), as reported by the Global Carbon Project [23]. This value is comparable to the carbon sink of 0.08–0.36 Pg C yr^−1^ in the USA [24,25] and 0.14–0.26 Pg C/yr in Europe [26,27]. All three models used in this study revealed an increasing NEP trend during 2000−10, albeit with different rates (range: 11.94–18.16 Tg C/yr^2^). This shift in NEP trend still exists whether we started the first decade of the twenty-first century with 1999, 2000 or 2001. We further examined the values of the NEP trend in China calculated from the global NEP estimation using different approaches and found that they consistently supported our results (see Supplementary Text 2 and Supplementary Fig. 1, available as Supplementary Data at *NSR* online). For example, the NEP values in China that were derived from the outputs of three process-based global ecosystem models, CLM4, CABLE and ORCHIDEE [28], showed an increasing trend of 14.66, 9.65 and 4.38 Tg C/yr^2^, respectively. The driving meteorological data of these global models from the CRU-NCEP had strong correlations with those of the models in this study (Supplementary Fig. 6, available as Supplementary Data at *NSR* online), so our modeling results are comparable to those of the three global models. Moreover, the gridded carbon-flux data in China, which was extracted from the up-scaled global carbon-flux data using eddy-covariance observations [29], exhibited a NEP acceleration of 16.63 Tg C/yr^2^. Furthermore, the top-down atmospheric inversions of CO_2_ surface fluxes from the European Centre for Medium-Range Weather Forecasts [30] and the CarbonTracker of CO_2_ measurement and modeling system [31] also produced a NEP upward trend of 14.08 and 16.18 Tg C/yr^2^ in China, respectively. The increasing trend of the modeled NEP in China during 2000–10 was also consistent with the global-scale acceleration of carbon uptake during the warming hiatus [11,13]. This upward trend in China's terrestrial NEP accounted for 11.6% of the global carbon uptake acceleration (122.73 Tg C/yr^2^ reported by Global Carbon Project) [23].

Our findings suggest that the mechanisms that triggered the carbon-sink enhancement in China were different from the global findings [11,13]. We found that, in addition to the warming hiatus, the strengthening East Asian summer monsoon was another critical climate factor that enhanced the carbon uptake in China during 2000–10. The East Asian summer monsoon is a large-scale atmospheric circulation pattern that plays a major role in regulating precipitation over a vast area of China. Previous studies have shown that the East Asian summer monsoon has become weak since the end of the 1970s but has been recovering in the last decade toward northward-moving rainbands and excessive rainfall in northern China, although its strength is still less than that in previous decades [7,32–34]. The strengthening summer monsoon caused an increase in precipitation in North China over the last decade, particularly in the temperate monsoonal climatic region (see Supplementary Fig. 2a and 2b, available as Supplementary Data at *NSR* online). Our modeled NEP in this region showed a high sensitivity to precipitation (Table 2), which is a key limiting factor of vegetation photosynthesis in the growing season [35,36]. Thus, the change in monsoonal precipitation from a decreasing to an increasing trend in this region (Fig. 5d) stimulated the ecosystem carbon uptake (see Supplementary Fig. 2c, available as Supplementary Data at *NSR* online). In addition, the warming hiatus suppressed the decrease in the NEP because of the larger deceleration of the RE increase than of the GPP increase, particularly in the subtropical–tropical monsoonal climatic region (Fig. 5b). In this region, the sensitivity of RE to temperature is much higher than that of GPP [37,38], which in turn led to the close association of NEP with temperature (Table 2). Therefore, more carbon gains that were driven by the enhanced summer monsoon and fewer carbon losses that were driven by the warming hiatus altered the trend in the carbon uptake of China's terrestrial ecosystems.

In addition to climate, other environmental factors may also influence ecosystem carbon uptake in China. For example, both nitrogen deposition and atmospheric CO_2_ concentration could enhance the NEP. Nitrogen deposition could stimulate photosynthesis via increasing the foliar nitrogen of plants [39] and canopy leaf area [28], thus enhancing the carbon sink. The nitrogen deposition in China during 2000–10 had a significant positive effect on the terrestrial NEP (see Supplementary Fig. 3, available as Supplementary Data at *NSR* online). This effect appeared to be especially strong in the subtropical monsoonal area, where the nitrogen-deposition rate was relatively high [20,40]. The rising atmospheric CO_2_ concentration over the past few decades has prompted more photosynthesis than respiration, thus leading to an increase in carbon sequestration in China [4,14,28] and throughout the world [11,41]. But these two factors contributed much less to the shift in the NEP trend than did climate (Fig. 6b and c), as revealed by modeled results from simulation Experiments II, III and IV (see Supplementary Table 3, available as Supplementary Data at *NSR* online) in our study. Previous studies on the effect of land-cover change on the carbon budget in China have shown large uncertainties in terms of both methods and land-cover data [3,14,42,43]. Our sensitivity analyses (simulation Experiment V in Supplementary Table 3, available as Supplementary Data at *NSR* online) suggested that land-cover change had a minor effect on China's terrestrial carbon sink (Fig. 6d), which might have been underestimated, since the input data LAI and FPAR used in BEPS and TEC contained some information of land-cover change. The effect of land-cover legacy on carbon uptake remained uncertain due to the scarcity and uncertainty of the historical land-cover data [44], although it may have affected the initial carbon stock [14]. The young forest age structure consistently facilitated the carbon uptake during the periods of 1982–2000 and 2000–10 [20, 45]; thus, it played a minor role in shifting the NEP trend. Overall, it is clear that the consideration of the concurrent changes in non-climate factors did not undermine the importance of the enhanced summer monsoon and the warming hiatus in shifting the NEP trend from the period of 1982–2000 to 2000–10.

To the best of our knowledge, this study provides the first attempt to understand the role of climate changes in shifting the trend in terrestrial NEP of China in recent decades. Our results showed that the enhanced East Asian summer monsoon could stimulate carbon uptake in water-limited ecosystems by increasing precipitation in North China and the warming hiatus could cause a slowdown in the NEP downward trend in areas where the NEP was negatively correlated with temperature. This finding indicates that the enhanced Asian summer monsoon played a critical role in the acceleration of carbon uptake in China. Our study provides some insight for carbon sequestration analyses in other monsoonal areas worldwide. Furthermore, the East Asian summer monsoon has been projected to enhance in the future [46,47]. The *IPCC Fifth Assessment Report* indicated that the monsoonal precipitation will increase, as revealed by most (>85%) CMIP5 models [48], although the probability of the persistence of the warming hiatus is lower than 25% [49,50]. This uncertainty poses a challenge to the prediction of the future carbon sequestration trend in China's terrestrial ecosystems.

## MATERIALS AND METHODS

### Model description

We used three models, namely CEVSA2 [40], BEPS [51] and TEC [52] (see Supplementary Text 1 and Supplementary Table 1, available as Supplementary Data at *NSR* online), to simulate the spatial and temporal variations in terrestrial NEP in China. These models have been well validated at the site scale in previous studies (see Supplementary Table 2, available as Supplementary Data at *NSR* online) and have been widely used to estimate carbon and water fluxes on regional and global scales, especially in China [4,40,51–53]. In this study, all models used the same driving data, including climate, land cover and soil texture. The ensemble mean of carbon fluxes simulated by these models was used to analyse the changes in terrestrial NEP trend in China during 1982–2010.

### Model input data

Climate data were interpolated from the observations at 1098 meteorological stations [54]. Land-cover data were generated from the ChinaCover dataset [55]. Soil-texture data were retrieved from the Food and Agriculture Organization's Harmonized World Soil Database (FAO HWSD). FPAR and LAI were satellite-derived Global Inventory Modeling and Mapping Studies (GIMMS) FPAR3g [56] and Global Mapping (GLOBMAP) LAI datasets [57]. Nitrogen-deposition data were produced through relating nitrogen deposition to precipitation, nitrogen fertilizer and fuel consumption [40]. Atmospheric CO_2_ concentration data were from the Mauna Loa CO_2_ dataset and historical CO_2_ dataset (http://www.co2.earth). For details, see Supplementary Text 3, available as Supplementary Data at *NSR* online.

### Model evaluation

To make the modeled carbon-pool sizes consistent with new observations, we used inventory data of vegetation and soil carbon density from 11 984 plots (Fig. 1a) collected from the field survey data across three natural vegetation groups (forests, grasslands and shrublands) in China [18] to adjust the carbon pools after spin-up for CEVSA2 and TEC and to calibrate the potential decomposition rates of the slow and passive soil organic carbon pools for BEPS. The eddy-covariance flux data, collected at five forest sites, five grassland sites and one cropland site (74 site years) of ChinaFLUX (Fig. 1a; see Supplementary Table 4, available as Supplementary Data at *NSR* online), were used to validate the modeled carbon fluxes at the grid cell level. Compared with the flux measurements, the mean values of the modeled GPP, RE and NEP explained 69, 74 and 38%, respectively, of the seasonal variations in the observed fluxes on average (Fig. 1b; see Supplementary Fig. 4, available as Supplementary Data at *NSR* online). Moreover, the means of the modeled vegetation and soil carbon-pool sizes were consistent with the inventory data (Fig. 1c and d; see Supplementary Fig. 5, available as Supplementary Data at *NSR* online) and the modeled total national carbon stocks in vegetation and soil were consistent with other studies (see Supplementary Table 5, available as Supplementary Data at *NSR* online).

### Model simulation experiments

To quantify the effects of climate, atmospheric CO_2_ concentration, nitrogen deposition and land cover on the shift of the terrestrial NEP trend in China, we designed and conducted five simulation experiments (see Supplementary Table 3, available as Supplementary Data at *NSR* online). In Experiment I, the models were driven by a constant CO_2_ concentration and a long-term averaged climate. Experiment II was the same as Experiment I, but it was driven by realistic climate data. The difference between the modeled NEP in these two experiments represented the effect of climate change on terrestrial NEP. Experiment III was the same as Experiment II, but it was driven by the time-varying CO_2_ data. The CO_2_ fertilization effect was defined by the difference between the modeled NEP in Experiments II and III. Experiment IV was the same as Experiment III but included nitrogen-deposition data. The effect of nitrogen deposition on terrestrial NEP was defined by the difference between the modeled NEP in Experiments III and IV. Experiment V was designed to investigate the effect of land cover on the NEP by conducting a sensitivity analysis using land-cover data in 1990, 2000 and 2010.

### Response of carbon fluxes to climate variables

We estimated the responses of GPP, RE and NEP to precipitation and temperature over the past three decades using a multiple regression approach [58]:

(1) \begin{equation*} y'\, = \,{\delta ^{{\mathop{\rm int}} }} \times P'\, + \,{\gamma ^{{\mathop{\rm int}} }} \times T' + \varepsilon \end{equation*}

where *y'* is the detrended anomaly of the ensemble mean of modeled GPP, RE and NEP; *P'* and *T'* are the detrended anomalies of precipitation and temperature, respectively; *δ^int^* and *γ^int^* represent the sensitivity of carbon fluxes to climate variables; and *ϵ* is the residual error.

### Contribution of precipitation and temperature to the NEP trends

The NEP in all climatic regions except the high-cold Tibetan Plateau climatic region had significant linear relationships with precipitation and temperature (Equation 2; see Supplementary Table 6, available as Supplementary Data at *NSR* online). Thus, the contributions (*f*_i_) of precipitation (*X*_P_) and temperature (*X*_T_) to the trend of NEP (*Y*) (Equation 3) are defined as the rate of the product of the predictor variable (i.e. *X*_i_) and its regression coefficient (i.e. *b*_i_) in the multiple linear regression equation (Equation 2):

(2) \begin{equation*} Y\, = \,{b_0} + {b_P} \times {X_P} + {b_T} \times {X_T} + \varepsilon \end{equation*}

(3) \begin{equation*} {f_i} = d\left( {{b_i} \times {X_i}} \right)/\,dt,i = P,T\end{equation*}

where *Y* is the mean NEP modeled by CEVSA2, BEPS and TEC; *t* is the year from 1982 to 2010; *b*_i_ is the regression coefficient for precipitation and temperature; *b_0_* is the constant term; and *ϵ* is the residual error.

## Supplementary Material

nwz021_Supplemental_File
