# Supplementary material for: Altered trends in carbon uptake in China's terrestrial ecosystems under the enhanced summer monsoon and warming hiatus
Source: Natl Sci Rev. 2019 Feb 17;6(3):505–14. doi: 10.1093/nsr/nwz021 (PMC8291462; doi:10.1093/nsr/nwz021)
Supplement: nwz021_Supplemental_File [file nwz021_supplemental_file.docx]

**Supplementary Data**

**Altered trends in carbon uptake in China’s terrestrial ecosystems under the enhanced summer monsoon and warming hiatus**

Honglin He^a,b#^, Shaoqiang Wang^a,b#^, Li Zhang^a,b^, Junbang Wang^a^, Xiaoli Ren^a^, Lei Zhou^a^, Shilong Piao^c,d^, Hao Yan^e^, Weimin Ju^f^, Fengxue Gu^g^, Shiyong Yu^h^, Yuanhe Yang^i,j^, Miaomiao Wang^a,j^, Zhongen Niu^a,j^, Rong Ge^a,j^, Huimin Yan^a,b^, Mei Huang^a^, Guoyi Zhou^l^, Yongfei Bai^i^, Zongqiang Xie^i^, Zhiyao Tang^k^, Bingfang Wu^m^, Leiming Zhang^a,b^, Nianpeng He^a,b^, Qiufeng Wang^a,b^, Guirui Yu^a,b1^

^a^ Synthesis Research Center of China’s Ecosystem Research Network, Key Laboratory of Ecosystem Network Observation and Modeling, Institute of Geographic Sciences and Natural Resources Research, Chinese Academy of Sciences, Beijing 100101, China

^b^ College of Resources and Environment, University of Chinese Academy of Sciences, Beijing 100190, China

^c^ Sino-French Institute for Earth System Science, College of Urban and Environment Sciences, Peking University, Beijing 100871, China

^d^ Key Laboratory of Alpine Ecology and Biodiversity, Institute of Tibetan Plateau Research, CAS Center for Excellence in Tibetan Plateau Earth Science, Chinese Academy of Sciences, Beijing, 100085, China

^e^ National Meteorological Center, China Meteorological Administration, Beijing 100081, China

^f^ International Institute for Earth System Science and Jiangsu Provincial Key Laboratory of Geographic Information Science and Technology, Nanjing University, Nanjing 210023, China

^g^ Key Laboratory of Dryland Agriculture, MOA, Institute of Environment and Sustainable Development in Agriculture, Chinese Academy of Agricultural Sciences, Beijing 100081, China

^h^ School of Geography, Geomatics, and Planning, Jiangsu Normal University, Xuzhou 221116, China

^i^ State Key Laboratory of Vegetation and Environmental Change, Institute of Botany, The Chinese Academy of Sciences, Beijing 100093, China

^j^ University of Chinese Academy of Sciences, Beijing 100049, China

^k^ Department of Ecology, College of Urban and Environmental Science, and Key Laboratory for Earth Surface Processes of the Ministry of Education, Peking University, 100871 Beijing, China

^l^ Key Laboratory of Vegetation Restoration and Management of Degraded Ecosystems, South China Botanical Garden, Chinese Academy of Sciences, Guangzhou 510650, China

^m^ Institute of Remote Sensing and Digital Earth, Chinese Academy of Sciences, Beijing 100094, China

**Supplementary Data**

**Supplementary Texts S1–S3**

**Supplementary Figures S1–S6**

**Supplementary Tables S1–S6**

**Supplementary Texts**

**Text S1. Model description**

We used the three models (i.e., CEVSA2, BEPS, and TEC) to quantify changes in gross primary production (GPP), ecosystem respiration (RE) and net ecosystem production (NEP) from 1982 to 2010. The model characteristics were summarized in Table S1.

**CEVSA2** is a process-based biogeochemical model developed from the Carbon Exchange between Vegetation, Soil, and the Atmosphere (CEVSA) model [1-5]. This model estimates carbon fluxes by integrating the processes of plant photosynthesis and respiration, stomata conductance, nitrogen uptake, evapotranspiration, carbon allocation among plant organs, litter production, nitrogen mineralization, and soil organic carbon decomposition. It has been widely used to analyze regional and global terrestrial carbon exchange and its response to climate changes [1, 2, 5-8]. Photosynthesis was simulated for each leaf layer of the canopy using the Farquhar model coupled to the modified Ball-Berry model by considering the regulation of soil moisture on the stomatal conductance [9]. A portion of photosynthate was consumed in autotrophic respiration, which was the sum of the maintenance respiration and growth respiration. Net primary production (NPP), the difference between the gross photosynthesis and autotrophic respiration, was allocated proportionally among leaves, stems, and roots. In this model, soil organic matter was divided into surface litter, root litter, microbes, and slow and passive carbon pools. The carbon decomposition in each pool was simulated by first-order rate reactions [10-12]. Major interactions between carbon and nitrogen, such as the effects of nitrogen on photosynthesis, respiration, carbon allocation, and soil carbon decomposition, was included in the CEVSA2 model [13], which has been successfully used to examine the effect of nitrogen deposition on carbon storage in forests [14].

For model spin-up, the CEVSA2 model was driven by the average climate status during 1951–1981 and atmospheric CO_2_ concentration in 1950 and run until an equilibrium was reached, i.e. the differences between annual NPP, litter production, and soil respiration, and the inter-annual variations in soil moisture, carbon storage in vegetation and soil are less than 0.1% [14]. The transient simulations were then driven by historical climate data and atmospheric CO_2_ concentration from 1951 to 2010.

**BEPS** (Boreal Ecosystem Productivity Simulator) is a biogeochemical model that simulates water, carbon, and nitrogen cycles in the terrestrial ecosystem using remotely sensed LAI as input [15, 16]. The Farquhar model was employed to simulate photosynthesis in the canopy, which was divided into sunlit and shaded leaves. The carbon and water cycle is linked by stomata conductance calculated using the ‘Jarvis’ model [17]. Soil organic carbon decomposition was simulated using a modified version of the CENTURY model [11, 18]. Soil heterotrophic respiration was expressed as a function of soil carbon mass, decomposition rate, and respiration efficiency of four soil carbon pools and five litter carbon pools. The model implicitly reflected the effects of human activities (such as land use change, human disturbance, nitrogen deposition, fertilization and irrigation) on the carbon cycle because of the incorporation of the remote sensing information. BEPS has been improved continuously and it has been widely used to estimate terrestrial carbon and water fluxes in China [19], North America [20, 21], Europe [22], East Asia [23], and the globe [24].

BEPS uses a spin-up scheme that is similar to the semi-analytical scheme developed by Xia et al. [25]. The model calculates the stable state based on the equilibrium hypothesis (i.e., NEP ≈ 0) using forcing data from 1901 to 1981. The daily meteorological data in 1950–2011 were interpolated from observations and those in 1901–1949 were annually randomly selected from the data in the period 1950–2000. The LAI data during 1901–1981 were annually randomly selected from the period 1982–2000. The transient simulations were driven by historical climate data, atmospheric CO_2_ concentration, and LAI from 1901 to 2011.

**TEC** (Terrestrial Ecosystem Carbon flux) is an integration of a remote-sensing based production efficiency model and the soil organic matter decomposition module of the CENTURY model. TEC can simulate carbon and water fluxes between the atmosphere and land surface at a monthly step. Gross primary production (GPP) was estimated by taking into account temperature and water stresses [26]. Water stress was calculated from the ratio of actual evaporation to potential evaporation with inputs of remotely sensed LAI and surface meteorological data [27]. Heterotrophic respiration and soil carbon dynamics were based on the CENTURY model. The carbon pools include five compartments such as leaves, fine roots, fine branches, large wood, and coarse roots, which are determined by NPP allocated to these different plant organs using a fixed allocation scheme and a constant turnover rate.

The TEC model was initialized to represent the storage and flux conditions at nearly steady state (i.e., inter-annual variations in carbon storage in vegetation and soil are less than 0.1%), driven by climate data in 1961–1981 and remotely sensed FPAR in 1981 [28]. Then the transient simulation was run using meteorological data from 1961 to 2011 and remotely sensed FPAR from 1961 to 2011. The FPAR data in 1961–1981 were substituted by the FPAR in 1982 because of lack of data.

**Text S2. NEP data from global flux estimation using different approaches**

To corroborate the overall increasing trend in terrestrial NEP in China during 2000–2010, we collected several independent estimates of carbon flux using different approaches, including bottom-up, top-down, and eddy covariance based upscaling. The NEP using the bottom-up approach was extracted from the global outputs of three process-based ecosystem models (CLM4, CABLE, and ORCHIDEE) [29]. The driving meteorological data of these global models from the CRU-NCEP had strong correlations with those of the models in this study (Fig. S7).

The CO_2_ surface fluxes using the top-down approach were derived from the outputs of atmospheric inversions in the European Centre for Medium-Range Weather Forecasts [30] (<http://apps.ecmwf.int/datasets/data/macc-ghg-inversions/>) and the CarbonTracker CO_2_ measurement and modeling system [31] (<https://www.esrl.noaa.gov/gmd/ccgg/carbontracker/>). The gridded NEP based on eddy covariance was derived from a global net ecosystem exchange data product, which was upscaled from FLUXNET observations of carbon dioxide using a machine learning technique [32].

**Text S3. Model input data**

**Climate data**, including temperature, precipitation, and relative humidity, were interpolated from the observations at 1098 stations across China using ANUSPLIN software [33, 34]. Incoming solar radiation was derived from an empirical relationship between global radiation and the duration of sunshine based on the Angstrom equation [35, 36]. The observations were obtained from the Chinese National Meteorological Information Center (http://data.cma.cn).

**FPAR** was a bimonthly product with a spatial resolution of 1/12 degree spanning the period from July 1981 to December 2011, which was produced using a trained neural network [37].

**LAI** was produced by fusing the Advanced Very High Resolution Radiometer (AVHRR) LAI (1981–2000) and MODIS LAI (2000–2010) products, which is also known as GLOBMAP LAI [38]. AVHRR LAI from 1981 to 2000 was estimated using the relationship between AVHRR observations and MODIS LAI at each pixel during the overlapping period of 2000–2006.

**Land cover data** was based on the ChinaCover dataset (including 1990, 2000, and 2010) [39], which was produced using Landsat TM/ETM and HJ-1 satellite data with a 30 m resolution combined with a large amount of data from field investigations. The accuracy of the ChinaCover data was validated using satellite images with a high spatial resolution and large amount of ground observational data. The distribution of each vegetation type was resampled to 8 km from the ChinaCover dataset [39].

**Nitrogen deposition data** were produced using a previously described algorithm[14], which relates nitrogen deposition to precipitation, nitrogen fertilizer use, and fuel consumption[14]. The parameters of the equations in this algorithm were fitted using observed dry and wet nitrogen deposition data collected from 163 sites across the country during 1985–2005. The gridded nitrogen deposition data (0.1°×0.1°) from 1951 to 2010 were generated based on gridded precipitation, county-level nitrogen fertilizer use, and province-level energy consumption.

**Soil data** included soil texture parameters determined from the fractions of clay, silt, and sand retrieved from the Food and Agriculture Organization’s Harmonized World Soil Database (FAO HWSD), which were used for the soil moisture simulations. The soil maps were compiled based on the second national soil survey using the standard system of soil genetic classification of China for delineating soil mapping units [40].

**Supplementary Figures**

**Fig. S1** Comparison of the trends of net ecosystem production (NEP) in China during 2000–2010 simulated from this study and other methods. “Global model” represents the results from three dynamic global vegetation models (CLM4, CABLE, and ORCHIDEE) [29]; “ Eddy covariance” represents the results from the global carbon flux data derived from the eddy covariance observations [41]; “Atmospheric inversion” represents the results from two top-down atmospheric inversions from the European Centre for Medium-Range Weather Forecasts (<http://apps.ecmwf.int/datasets/data/macc-ghg-inversions/>) and the CarbonTracker of CO_2_ measurement and modeling system (<https://www.esrl.noaa.gov/gmd/ccgg/carbontracker/>).

**Fig. S2** The change in East Asian Summer Monsoon Index (EASMI) during 2000–2010 (A) and its correlation with summer precipitation (B) and annual net ecosystem production (NEP) (C) in the temperate monsoonal region.

**Fig. S3** Relationship between the modeled net ecosystem production (NEP) and nitrogen deposition in China during 2000–2010.

**Fig. S4** Validation of gross primary production (GPP), ecosystem respiration (RE), and net ecosystem production (NEP) simulated by the CEVSA2, BEPS, and TEC models against the observational data at 11 flux tower sites. These sites are Yucheng cropland (YC), Dangxiong alpine steppe-meadow (DX), Haibei alpine shrub-meadow (HBGC), Inner Mongolia temperate steppe (NMG), Xilinguole grassland (XLGL), Haibei alpine swamp (HBSD), Dinghushan evergreen mixed forest (DHS), Changbaishan temperate mixed forest (CBS), Huitong evergreen needleleaf forest (HT), Qianyanzhou evergreen needleleaf forest (QYZ), Xishuangbanna evergreen broadleaf forest (XSBN), and all sites together (ALL). The goodness of fit was determined using the coefficient of determination (*R*^2^).

**Fig. S5** Validation of vegetation and soil carbon storages simulated by CEVSA2, BEPS, and TEC. (A) Comparison of the modeled and observed vegetation carbon storages in different vegetation types. (B) Comparison of the modeled and observed soil carbon storages in various vegetation types. The vegetation types are broadleaf evergreen forest (BEF), needleleaf evergreen forest (NEF), broadleaf deciduous forest (BDF), needleleaf deciduous forest (NDF), mixed forest (MF), shrubland (SH), and grassland (GRA). Error bars in panels (C) and (D) represent the standard error.

**Fig. S6** Comparison of (**A**) temperature and (**B**) precipitation data used in this study with those from the CRU-NCEP dataset.

**Supplementary Tables**

**Table S1.** Summary of the terrestrial carbon cycle models used in this study.

| Model characteristics | CEVSA2 | BEPS | TEC |
| --- | --- | --- | --- |
| Time step | 10 days | Daily | Monthly |
| Spatial resolution | 10 km | 8 km | 8 km |
| Simulation period | 1951−2010 | 1901−2011 | 1961−2011 |
| Phenology | Prognostic | Derived LAI from AVHRR and MODIS | AVHRR NDVI |
| Photosynthesis | Farquhar model, Big-leaf | Farquhar model, 2-Leaf | LUE |
| Soil carbon decomposition | 1^st^ order | 1^st^ order | 1^st^ order |
| Model inputs | Climate, CO_2_, soil, nitrogen deposition, and land use | Climate, CO_2_, soil, LAI, and land use | Climate, CO_2_, soil, LAI, FPAR, and land use |
| Model outputs | GPP, NPP, NEP, C pool sizes | GPP, NPP, NEP, and C pool sizes | GPP, NPP, NEP, and C pool sizes |
| References | [14] | [16] | [26] |

**Table S2.** Validation of the modeled carbon fluxes in previous studies.

| Model name | Site name | Vegetation type | Longitude  (°E) | Latitude  (°N) | Carbon flux | *R*^2^ | Slope | Reference |
| --- | --- | --- | --- | --- | --- | --- | --- | --- |
| CEVSA2 | Harvard forest | DBF | 72.17 | 42.53 | NEP | 0.76 | 0.8 | [13] |
|  | Changbaishan temperate mixed forest | MF | 128.09 | 42.40 | RE | 0.83 | 0.86 | [6] |
|  | Changbaishan temperate mixed forest | MF | 128.09 | 42.40 | NEP | 0.34 | 0.59 | [6] |
|  | Qianyanzhou evergreen needle leaf forest | ENF | 115.07 | 26.75 | GPP | 0.88 |  | [42] |
|  |  |  |  |  | RE | 0.94 |  |  |
|  |  |  |  |  | NEP | 0.52 |  |  |
|  | Changbaishan temperate mixed forest | MF | 128.09 | 42.40 | GPP | 0.94 |  |  |
|  |  |  |  |  | RE | 0.97 |  |  |
|  |  |  |  |  | NEP | 0.67 |  |  |
|  | Harvard forest | DBF | 72.17 | 42.53 | GPP | 0.96 |  |  |
|  |  |  |  |  | RE | 0.80 |  |  |
|  |  |  |  |  | NEP | 0.89 |  |  |
|  | National Forest Inventory data (420 plots) | forest | - | - | NPP | 0.61 | 0.68 | [7] |
|  | grassland observation data (335 sites) | grassland | - | - | NPP | 0.18 | 0.38 |  |
|  | Statistical data for 1246 counties | cropland | - | - | NPP | 0.14 | 0.40 |  |
| BEPS | Qianyanzhou evergreen needle leaf forest | NEF | 115.07 | 26.75 | GPP | 0.83 | 0.96 | [16] |
|  | Changbaishan temperate mixed forest | MF | 128.09 | 42.40 | GPP | 0.84 | 0.93 | [43] |
|  | Qianyanzhou evergreen needle leaf forest | ENF | 115.07 | 26.75 |  |  |  |  |
|  | Yucheng cropland | Crop | 116.64 | 36.96 |  |  |  |  |
|  | Haibei alpine shrub-meadow | Grass | 101.33 | 37.61 |  |  |  |  |
|  | Changbaishan temperate mixed forest | MF | 128.09 | 42.40 | GPP | 0.90 | 1.18 | [44] |
|  | Qianyanzhou evergreen needle leaf forest | ENF | 115.07 | 26.75 | GPP | 0.70 | 0.84 |  |
|  | Dinghushan evergreen broadleaved forest | EBF | 112.53 | 23.17 | GPP | 0.46 | 1.06 |  |
|  | Yucheng cropland | Crop | 116.64 | 36.96 | GPP | 0.62 | 0.70 |  |
|  | Haibei alpine meadow  meadow | Grass | 101.33 | 37.61 | GPP | 0.86 | 1.17 |  |
|  | Xinlinhot grassland | Grass | 116.67 | 43.55 | GPP | 0.58 | 0.48 |  |
|  | Qianyanzhou evergreen needle leaf forest | ENF | 115.07 | 26.75 | GPP | 0.86 | 1.09 | [45] |
|  | GPPDI dataset (http://www-eosdis.ornl.gov) |  |  |  | NPP | 0.78 | 0.78 | [46] |
| TEC | Bartlett experimental forest | DBF | –71.29 | 44.06 | GPP* | 0.72 | 0.73 | [26] |
|  | Metolius intermediate pine | ENF | –121.62 | 44.50 |  |  |  |  |
|  | Metolius new young pine | ENF | –121.61 | 44.32 |  |  |  |  |
|  | Mize | ENF | –82.24 | 29.76 |  |  |  |  |
|  | Morgan Monroe state forest | DBF | –86.41 | 39.32 |  |  |  |  |
|  | University of Michigan biological  station | DBF | –84.71 | 45.56 |  |  |  |  |
|  | Wind river crane site | ENF | –121.95 | 45.82 |  |  |  |  |
|  | ARM SGP main | Crop | –97.49 | 36.61 |  |  |  |  |
|  | Bondville | Crop | –88.29 | 40.01 |  |  |  |  |
|  | Fermi agricultural | Crop | –88.22 | 41.86 |  |  |  |  |
|  | Mead irrigated | Crop | –96.47 | 41.16 |  |  |  |  |
|  | Mead irrigated rotation | Crop | –96.47 | 41.16 |  |  |  |  |
|  | Mead rainfed | Crop | –96.43 | 41.17 |  |  |  |  |
|  | Fermi prairie | Grass | –88.24 | 41.84 |  |  |  |  |
|  | Barrow | Grass | –156.63 | 71.32 |  |  |  |  |
|  | Santa Rita mesquite savanna | Shrub | –110.87 | 31.82 |  |  |  |  |
|  | Tonzi ranch | Savanna | –120.97 | 38.43 |  |  |  |  |
|  | Vaira ranch | Savanna | –120.95 | 38.41 |  |  |  |  |

Note: DBF, Deciduous broadleaf forest; ENF, Evergreen needleleaf forest; EBF, Evergreen broadleaf forest; and MF, mixed forest.

* These sites were validated all together.

**Table S3.** Simulation experiments conducted in this study. The differences between the modeled NEP in experiment II and experiment I, experiment III and experiment II, experiment IV and experiment III represent the effects of climate, CO_2_, and nitrogen deposition on the terrestrial NEP, respectively. Experiment V was designed to explore the effect of land use on the modeled NEP by conducting sensitivity analysis using land use data in 1990, 2000, and 2010.

| Experiment | CO_2_ | Climate | Nitrogen deposition | Land use | Scenario |
| --- | --- | --- | --- | --- | --- |
| I | Constant^*^ | Constant^#^ | - | 2010 | Control |
| II | Constant^*^ | Transient | - | 2010 | Climate |
| III | Transient | Transient |  | 2010 | Climate+CO_2_ |
| IV | Transient | Transient | Transient | 2010 | Climate+CO_2_+nitrogen |
| V | Transient | Transient | Transient | 1990, 2000, 2010 | Sensitivity analysis of land use data |

^*^ CO_2_ concentration was fixed at the value in 1960 for CEVSA2 and BEPS.

^#^ Climate was the average climate during 1960–1969.

**Table S4.** Description of the eddy covariance flux tower sites used in this study.

| Vegetation  type | Site name | Climate  region | Longitude | Latitude | Start year | End year |
| --- | --- | --- | --- | --- | --- | --- |
| Cropland | Yucheng cropland (YC) | II | 116.64 | 36.96 | 2003 | 2010 |
| Grassland | Dangxiong alpine steppe-meadow (DX) | III | 91.07 | 30.50 | 2004 | 2010 |
|  | Haibei alpine shrub-meadow (HBGC) | III | 101.33 | 37.67 | 2003 | 2010 |
|  | Inner Mongolia temperate steppe (NMG) | I | 116.30 | 44.13 | 2004 | 2010 |
|  | XiLinGuoLe grassland (XLGL) | I | 116.67 | 43.53 | 2010 | 2010 |
|  | Haibei alpine swamp (HBSD) | III | 101.33 | 37.61 | 2003 | 2010 |
| Forest | Changbaishan temperate mixed forest (CBS) | II | 128.10 | 42.40 | 2003 | 2010 |
|  | Dinghushan evergreen mixed forest (DHS) | IV | 112.50 | 23.15 | 2003 | 2010 |
|  | Huitong evergreen needle leaf forest (HT) | IV | 109.59 | 26.79 | 2008 | 2010 |
|  | Qianyanzhou evergreen needle leaf forest (QYZ) | IV | 115.05 | 26.73 | 2003 | 2010 |
|  | Xishuangbanna evergreen broadleaf forest (XSBN) | IV | 101.27 | 21.90 | 2003 | 2010 |

Note: I, Temperate continental; II, Temperate monsoonal; III, Tibetan Plateau; and IV, Subtropical-tropical monsoonal.

**Table S5.** Comparison of the modeled and observed carbon storages for different vegetation and soil types (unit: Pg C/yr).

| Carbon stock | CEVSA2 | BEPS | TEC | Mean | Observation | Reference |
| --- | --- | --- | --- | --- | --- | --- |
| Vegetation | 23.23±0.58 | 27.71±0.48 | 24.10±0.16 | 25.00±0.35 | 6.1−76.2 | [47] |
| Forest | 13.28±0.33 | 21.13±0.35 | 18.44±0.10 | 17.61±0.23 | 10.48±2.02 | [48] |
| Shrub | 1.25±0.03 | 1.12±0.02 | 1.94±0.01 | 1.44±0.02 | 0.71±0.23 | [48] |
| Grassland | 2.50±0.05 | 0.98±0.03 | 1.12±0.04 | 1.53±0.03 | 1.35±0.47 | [48] |
| Soil | 99.52±0.24 | 41.10±0.34 | 66.31±0.31 | 68.97±0.27 | 93  43.6−185.7 | [49]  [47] |
| Forest | 29.99±0.20 | 19.62±0.16 | 33.74±0.15 | 27.78±0.17 | 19.98±2.41 | [48] |
| Shrub | 4.04±0.03 | 2.19±0.02 | 3.19±0.01 | 3.14±0.02 | 5.91±0.43 | [48] |
| Grassland | 35.33±0.26 | 6.78±0.05 | 9.46±0.06 | 17.19±0.06 | 24.03±2.52 | [48] |

**Table S6.** Results of multiple regression analysis between NEP and climate factors, i. e., temperature (TEM) and precipitation (PRC), for four climatic regions and throughout China.

| Climatic region | Regression equation | *R*^2^ | *P* value |
| --- | --- | --- | --- |
| Temperate continental (I) | NEP=0.63×PRC-8.39×TEM-122.17 | 0.82 | 0.000 |
| Temperate monsoonal (II) | NEP=0.57×PRC-8.93×TEM-255.64 | 0.74 | 0.000 |
| High-cold Tibetan Plateau (III) | NEP=0.01×PRC-1.18×TEM+7.78 | 0.01 | 0.598 |
| Subtropical-tropical monsoonal (IV) | NEP=0.16×PRC-27.95×TEM+336.67 | 0.19 | 0.017 |
| China | NEP=1.13×PRC-47.33×TEM-338.01 | 0.32 | 0.001 |

**References**

1. Cao M, Prince SD, Li K *et al.* Response of terrestrial carbon uptake to climate interannual variability in China. *Global Change Biology* 2003; **9**:536-46.

2. Cao M, Prince SDandShugart HH. Increasing terrestrial carbon uptake from the 1980s to the 1990s with changes in climate and atmospheric CO2. *Global Biogeochemical Cycles* 2002; **16**.

3. Cao M, Prince SD, Small J *et al.* Remotely sensed interannual variations and trends in terrestrial net primary productivity 1981–2000. *Ecosystems* 2004; **7**:233-42.

4. Cao MandWoodward FI. Net primary and ecosystem production and carbon stocks of terrestrial ecosystems and their responses to climate change. *Global Change Biology* 1998; **4**:185-98.

5. Cao MandWoodward FI. Dynamic responses of terrestrial ecosystem carbon cycling to global climate change. *Nature* 1998; **393**:249-52.

6. Tao B, Cao M, Li K *et al.* Spatial patterns of terrestrial net ecosystem productivity in China during 1981–2000. *Science in China Series D: Earth Sciences* 2007; **50**:745-53.

7. Gu F, Y Z, Huang M *et al.* Effects of climate warming on net primary productivity in China during 1961–2010. *Ecology and Evolution* 2017; **(accepted)**.

8. Cao M, Prince SD, Tao B *et al.* Regional pattern and interannual variations in global terrestrial carbon uptake in response to changes in climate and atmospheric CO2. *Tellus B* 2005; **57**:210-7.

9. Woodward FI, Smith TMandEmanuel WR. A global land primary productivity and phytogeography model. *Global biogeochemical cycles* 1995; **9**:471-90.

10. Parton W, Schimel DS, Cole C *et al.* Analysis of factors controlling soil organic matter levels in Great Plains grasslands. *Soil Science Society of America Journal* 1987; **51**:1173-9.

11. Parton W, Scurlock J, Ojima D *et al.* Observations and modeling of biomass and soil organic matter dynamics for the grassland biome worldwide. *Global biogeochemical cycles* 1993; **7**:785-809.

12. Parton WJ, Stewart JWandCole CV. Dynamics of C, N, P and S in grassland soils: a model. *Biogeochemistry* 1988; **5**:109-31.

13. Gu F, Zhang Y, Tao B *et al.* Modeling the effects of nitrogen deposition on carbon budget in two temperate forests. *Ecological Complexity* 2010; **7**:139-48.

14. Gu F, Zhang Y, Huang M *et al.* Nitrogen deposition and its effect on carbon storage in Chinese forests during 1981–2010. *Atmospheric Environment* 2015; **123**:171-9.

15. Ju W, Chen JM, Black TA *et al.* Modelling multi-year coupled carbon and water fluxes in a boreal aspen forest. *Agricultural and Forest Meteorology* 2006; **140**:136-51.

16. Ju W, Wang S, Yu G *et al.* Modeling the impact of drought on canopy carbon and water fluxes for a subtropical evergreen coniferous plantation in southern China through parameter optimization using an ensemble Kalman filter. *Biogeosciences* 2010; **7**:845-57.

17. Jarvis P. The interpretation of the variations in leaf water potential and stomatal conductance found in canopies in the field. *Philosophical Transactions of the Royal Society of London B: Biological Sciences* 1976; **273**:593-610.

18. Ju WandChen JM. Distribution of soil carbon stocks in Canada's forests and wetlands simulated based on drainage class, topography and remotely sensed vegetation parameters. *Hydrological Processes* 2005; **19**:77-94.

19. Feng X, Liu G, Chen J *et al.* Net primary productivity of China's terrestrial ecosystems from a process model driven by remote sensing. *Journal of environmental management* 2007; **85**:563-73.

20. Schwalm CR, Williams CA, Schaefer K *et al.* A model‐data intercomparison of CO2 exchange across North America: Results from the North American Carbon Program site synthesis. *Journal of Geophysical Research: Biogeosciences* 2010; **115**.

21. Sprintsin M, Chen JM, Desai A *et al.* Evaluation of leaf‐to‐canopy upscaling methodologies against carbon flux data in North America. *Journal of Geophysical Research: Biogeosciences* 2012; **117**.

22. Wang Q, Tenhunen J, Falge E *et al.* Simulation and scaling of temporal variation in gross primary production for coniferous and deciduous temperate forests. *Global Change Biology* 2004; **10**:37-51.

23. Matsushita BandTamura M. Integrating remotely sensed data with an ecosystem model to estimate net primary productivity in East Asia. *Remote Sensing of Environment* 2002; **81**:58-66.

24. Chen JM, Mo G, Pisek J *et al.* Effects of foliage clumping on the estimation of global terrestrial gross primary productivity. *Global Biogeochemical Cycles* 2012; **26**.

25. Xia J, Luo Y, Wang Y-P *et al.* A semi-analytical solution to accelerate spin-up of a coupled carbon and nitrogen land model to steady state. *Geoscientific Model Development* 2012; **5**:1259-71.

26. Yan H, Wang S-q, Billesbach D *et al.* Improved global simulations of gross primary product based on a new definition of water stress factor and a separate treatment of C3 and C4 plants. *Ecological Modelling* 2015; **297**:42-59.

27. Yan H, Wang SQ, Billesbach D *et al.* Global estimation of evapotranspiration using a leaf area index-based surface energy and water balance model. *Remote Sensing of Environment* 2012; **124**:581-95.

28. Potter C, Klooster S, Huete A *et al.* Terrestrial carbon sinks in the Brazilian Amazon and Cerrado region predicted from MODIS satellite data and ecosystem modeling. *Biogeosciences Discussions* 2009; **6**:937-45.

29. Piao S, Yin G, Tan J *et al.* Detection and attribution of vegetation greening trend in China over the last 30 years. *Global change biology* 2015; **21**:1601-9.

30. Chevallier F, Ciais P, Conway T *et al.* CO2 surface fluxes at grid point scale estimated from a global 21 year reanalysis of atmospheric measurements. *Journal of Geophysical Research: Atmospheres (1984–2012)* 2010; **115**.

31. Zhang H, Chen B, Laan‐Luijkx vdI *et al.* Net terrestrial CO2 exchange over China during 2001–2010 estimated with an ensemble data assimilation system for atmospheric CO2. *Journal of Geophysical Research: Atmospheres* 2014; **119**:3500-15.

32. Jung M, Reichstein M, Margolis HA *et al.* Global patterns of land-atmosphere fluxes of carbon dioxide, latent heat, and sensible heat derived from eddy covariance, satellite, and meteorological observations. *Journal of Geophysical Research: Biogeosciences* 2011; **116**:G00J7.

33. Hutchinson MF. ANUSPLIN Version 4.4 User Guide. *http://fennerschool.anu.edu.au/research/publications/software-datasets/anusplin* 2004.

34. Wang J, Wang J, Ye H *et al.* An interpolated temperature and precipitation dataset at 1-km grid resolution in China (2000-2012). *China Scientific Data* 2017; **2**.

35. Zhu XD, He HL, Liu M *et al.* Spatio-temporal variation of photosynthetically active radiation in China in recent 50 years. *Journal of Geographical Sciences* 2010; **20**:803-17.

36. Ren XL, He HL, Zhang L *et al.* Spatiotemporal variability analysis of diffuse radiation in China during 1981–2010. *Ann. Geophys.* 2013; **31**:277-89.

37. Zhu Z, Bi J, Pan Y *et al.* Global Data Sets of Vegetation LAI3g and FPAR3g derived from GIMMS NDVI3g for the period 1981 to 2011. *Remote Sens* 2013; **5**:927-48.

38. Liu Y, Liu RandChen JM. Retrospective retrieval of long‐term consistent global leaf area index (1981–2011) from combined AVHRR and MODIS data. *Journal of Geophysical Research: Biogeosciences* 2012; **117**.

39. Wu BF, Wang ZM, Zeng Y *et al.* Land cover in China: changes, drivers and implications. *P Natl Acad Sci USA* 2017.

40. Shi XZ, Yu DS, Warner ED *et al.* Soil Database of 1:1,000,000 Digital Soil Survey and Reference System of the Chinese Genetic Soil Classification System. *Soil Survey Horizons* 2004; **45**:129.

41. Jung M, Reichstein M, Margolis HA *et al.* Global patterns of land‐atmosphere fluxes of carbon dioxide, latent heat, and sensible heat derived from eddy covariance, satellite, and meteorological observations. *Journal of Geophysical Research: Biogeosciences* 2011; **116**.

42. Gu F, Cao M, Yu G *et al.* Modeling carbon exchange in different forest ecosystems by CEVSA model: Comparison with eddy covariance measurements. *Advances in Earth Science* 2007; **22**:313-21. (in Chinese).

43. Liu Y, Ju W, He H *et al.* Changes of net primary productivity in China during recent 11 years detected using an ecological model driven by MODIS data. *Frontiers of Earth Science* 2013; **7**:112-27.

44. He M, Ju W, Zhou Y *et al.* Development of a two-leaf light use efficiency model for improving the calculation of terrestrial gross primary productivity. *Agricultural & Forest Meteorology* 2013; **173**:28-39.

45. Li D, Ju W, Lu D *et al.* Impact of estimated solar radiation on gross primary productivity simulation in subtropical plantation in southeast China. *Solar Energy* 2015; **120**:175-86.

46. Zhang F, Ju W, Shen S *et al.* Variations of Terrestrial Net Primary Productivity in East Asia. *Terrestrial, Atmospheric & Oceanic Sciences* 2012; **23**.

47. Ni J. Carbon storage in Chinese terrestrial ecosystems: approaching a more accurate estimate. *Climatic Change* 2013; **119**:905-17.

48. Tang X, Zhao X, Yongfei B *et al.* Carbon pools in China’s terrestrial ecosystems: new estimates based on an intensive field survey. *P Natl Acad Sci USA* 2017.

49. Wang S, Tian H, Liu J *et al.* Pattern and change of soil organic carbon storage in China: 1960s–1980s. *Tellus B* 2003; **55**:416-27.
